# Southern Tunisia: A still high endemicity area for hepatitis A

**DOI:** 10.1371/journal.pone.0175887

**Published:** 2017-04-20

**Authors:** Houcine Neffatti, Patricia Lebraud, Corinne Hottelet, Jawher Gharbi, Taieb Challouf, Anne-Marie Roque-Afonso

**Affiliations:** 1 AP-HP, Hôpital Paul Brousse, Virologie, Villejuif, France; 2 Université de Monastir, Institut Supérieur de Biotechnologie, Monastir, Tunisia; 3 Hôpital de Médenine, Médecine interne, Medenine, Tunisia; 4 INSERM U1193, Villejuif, France; CEA, FRANCE

## Abstract

**Background:**

Hepatitis A (HAV) and E (HEV) viruses are responsible for enterically transmitted hepatitis. Tunisia is reported to be of intermediate endemicity for HAV and of low seroprevalence for HEV; however, data from rural areas of South Tunisia are lacking.

**Methods:**

Sera from 216 asymptomatic pregnant women and from 92 patients with acute hepatitis were collected between October 2014 and November 2015. Total and IgM anti-HAV immunoglobulins and anti-HEV IgG and IgM were investigated. Anti-HAV IgM-positive samples were subjected to RT-PCR targeting the VP1/2A region and sequenced. HEV IgM positive samples and all samples from acute hepatitis patients were assessed for HEV RNA.

**Results:**

Among pregnant women (mean age 32+/-8), HAV seroprevalence was 98.6%, none presented anti-HAV IgM; HEV seroprevalence was 5.1% and three presented weakly reactive anti-HEV IgM without detectable RNA. Among acute hepatitis patients (mean age 18.5 +/- 14), HEV seroprevalence was 19,5%, none presented anti-HEV IgM, nor HEV RNA. HAV seroprevalence exceeded 90% by age 5 and acute HAV infection was detected in 20 patients (21,7%), younger than patients with other hepatitis causes (9,8 years vs. 20,4 years, p = 0,004); 65% were male. Most acute HAV infections were observed in a coastal area where HAV infections represented 52% of hepatitis etiology. Phylogenetic analysis identified genotype IA strains, clustering close to previously published Tunisian sequences.

**Conclusion:**

The present study confirmed a low HEV endemicity and evidenced a still high level of HAV circulation in Southern Tunisia, suggesting distinct dissemination patterns for these viruses.

## Introduction

Hepatitis A (HAV) and hepatitis E virus (HEV) are the main causes of acute viral hepatitis worldwide. Both are enterically transmitted naked RNA viruses that can cause outbreaks or sporadic cases.

HAV is transmitted through the fecal-oral route from person to person or via ingestion of contaminated food or water. Prevalence and incidence are strongly correlated with socioeconomic indicators and inadequate sanitation. In developing countries, particularly in rural areas, Hepatitis A is a disease of early childhood: prevalence exceeds 90% by age 10 and most infections are asymptomatic. As incomes rises and access to clean water and sanitation increases, age of infection shifts upward and the clinical manifestations become more severe. Over 100 million of individuals are estimated to become infected with HAV each year [1–3]

HEV has two different modes of spreading according to genotypes. Genotypes 1 and 2, which exclusively infect humans, are spread by the fecal-oral route and are responsible for waterborne outbreaks and sporadic cases in developing countries with poor sanitary conditions. Estimates of over 20 million infections per year have been published for these genotypes with significant mortality among pregnant women [4]. The burden of HEV genotype 3 and 4, that infect both humans and several mammal species, is less understood. Domestic pigs and wild boars have been recognized as the main animal reservoirs, although other animal species such as deer, rabbits and dromedaries may also serve as reservoirs. Reported transmission modes are ingestion of contaminated food or contact with infected animals but environmental contamination may also play a role. These zoonotic genotypes are responsible for acute sporadic cases in both developed and developing countries. Most infections are asymptomatic, but a chronic course may be observed among immunosuppressed patients with development of liver fibrosis [5].

A number of studies have described the prevalence of enterically transmitted hepatitis viruses in various parts of Tunisia [6] [7–9]. However, there are no data from the Southern governorates. Therefore, the present study was conducted to evaluate the disease burden of hepatitis A and E viruses in this region.

## Materials and methods

### Samples

Participants were included from October 2014 to November 2015 from three governorates of Southern Tunisia: Gabès, Medenine and Tataouine. These governorates cover 55,221 km^2^, with an estimated population density around 60 per km^2^ in 2014. Gabès and Medenine governorates are the most populated with respectively 374,300 and 479,520 inhabitants, while Tataouine has only 149,453 inhabitants and a population density of less than 4/km^2^. The climate is dry and rain occurs between October and January. Gabès and Medenine are coastal regions with agricultural and livestock activities. Tataouine is a vast governate of which nearly 90% of the land is desert, with herds and camels. In general, water for drinking is supplied during rainfall period and more than 50% of houses are not connected to collective sanitation plants

Sera were collected from 2 distinct groups: 1/ 216 blood samples were collected from apparently healthy pregnant women during routine blood tests as part of antenatal care in the hospital of Medenine 2/ 92 blood samples were collected from patients with a clinical diagnosis of acute hepatitis and elevated aminotransferases levels (ALT>50UI/L). Patients with evident chronic hepatitis, toxic hepatitis, or alcohol or metabolism disorders were excluded.

Pregnant women and patients with acute hepatitis were consecutive outpatients from the 3 medical structures. They were systematically invited to participate by the physicians involved in the study. The study procedures were explained to patients or to the parents of each child and oral informed consents were obtained. Consent was documented in each individual medical file by physicians in charge of the patients. The study protocol was reviewed by the Ethical Review Committee of the hospital of Medenine (reference HAE2015).

### Virological analysis

Sera were tested for IgG and IgM anti-HEV with Wantai HEV-IgG ELISA and Wantai HEV-IgM ELISA kits, respectively (Wantai Biologicals, Beijing, China) and for total (IgG+IgM) antibodies and IgM anti-HAV with ETI-AB-HAVK PLUS and ETI-HA-IGMK PLUS kits, respectively (Diasorin, Saluggia, Italy). For HEV IgG and IgM, assay results with index > 1.3 were considered as positive; specimens with equivocal results (index 0.7–1.3) were considered as negative. Anti-HAV IgG avidity was performed as previously described [10].

HEV RNA was tested by real-time RT-PCR assay (Altona Diagnostic Technologies, Hamburg, Germany). HAV RNA was tested by an in-house RT-PCR assay as described [11]. The assay targets a 508 base-pair fragment encompassing the VP1/2A junction which is further subjected to sequencing. Phylogenetic analysis was conducted in MEGA6 software with the Neighbor-Joining method from a Kimura 2-parameter distance matrix. Genotype was determined by using reference sequence belonging to different HAV genotypes (GenBank accession numbers X75215, AB020567, AB020564 and AF357222 for IA; M14707 and M20273 for IB; AY644676 for IIA; AY644670 for IIB; AY644337 and AJ299464 for IIIA and D00924 for V).

### Statistical analysis

Categorical variables were compared with Fisher’s exact test or Pearson Chi square test and continuous variables by Student’s t-test or Mann-Whitney U test, as appropriate, using Analyse-it 4.65 software. Statistical significance was set at p < 0.05.

## Results

### Pregnant women group

All pregnant women were out-patients of the Medenine hospital. The mean age was 32 ± 5 years (range 19–46) and the mean gestational time was 16 ± 8 weeks. HEV and HAV seroprevalence was 5.1% and 98.6%, respectively Table 1.

**Table 1:** HAV and HEV markers among pregnant women from Medenine by age group.

			HAV			HEV		
		Overall	Total Ig N (%)	IgM N	RNA N	IgG N (%)	IgM N	RNA N
**Age group** (years)	19–25	27	26 (94.6%)	0	0	1 (3.7%)	0	0
	26–30	65	63 (96.9%)	0	0	1 (1.5%)	1 (1.5%)	0
	31–35	62	62 (100%)	0	0	4 (6.4%)	2 (3.2%)	0
	36–40	42	42 (100%)	0	0	3 (7.1%)	0	0
	>40	11	11 (100%)	0	0	2 (18.2%)	0	0
**Total**		216	212 (98.6%)	0	0	11 (5.1%)	3 (1.4%)	0

The age of anti HEV-positive women was not different from that of HEV-negative women (33,6 vs. 31,9 years, p = 0,29). None tested positive for anti-HAV IgM. Positive anti-HEV IgM, with low IgM index (<3) and without detectable HEV RNA, were found in 3 patients, of whom 2 were also positive for anti-HEV IgG. These three women were 27, 31 and 35-year-old, with gestational times of 5–6 months and normal aminotransferases levels. All 11 women with anti-HEV IgG-positive results had markers of past HAV infection S1 Table.

### Acute hepatitis group

Ninety-two patients admitted for acute hepatitis to medical centers of the Southern Tunisian governorates were included: 25 from Gabès, 40 from Medenine, and 27 from Tataouine. Fifty-six percent were male and the mean age was 18+/-22 years (range 1–62). Patients with acute hepatitis from Gabès were younger than patients from the other governorates (12.5+/-11.7 years-old vs 22.3+/-16 in Medenine and 17.1+/-14 in Tataouine, p = 0.032).

HEV seroprevalence was 19,5%, ranging from 5,4% in patients under 10 years-old to 33,3% in patients over 40 years-old, but appears to reach a steady state around 30% above 10 years old. Anti-HEV IgG-positive patients were older than HEV-negative patients (25,8 vs. 16,2 years, p = 0,0143) with no difference according to sex (17,3% vs 22,5% for males and females, respectively, p = 0.6), or governorate (12%, 22.2% and 22.5% in Gabès, Medenine, and Tataouine, respectively; p = 0.53). None tested positive for anti-HEV IgM. HEV RNA was also undetectable in all 92 patients Table 2.

**Table 2:** HAV and HEV markers among patients with acute hepatitis by age group, gender and study region.

			HAV			HEV		
		Overall	Total Ig N (%)	IgM N (%)	RNA N (%)	IgG N (%)	IgM N	RNA N
**Age group** (years)	1–10	37	35 (94.6%)	14 (37.8%)	14 (37.8%)	2 (5.4%)	0	0
	11–20	20	19 (95.0%)	4 (20.0%)	3 (15.0%)	6 (30.0%)	0	0
	21–30	11	10 (90.9%)	2 (18.2%)	1 (9.1%)	3 (27.3%)	0	0
	31–40	15	15 (100%)	3 (20.0%)	2 (13.3%)	4 (26.7%)	0	0
	>40	9	9 (100%)	0	0	3 (33.3%)	0	0
**Sex**	Male	52	49 (94.2%)	14 (26.9%)	13 (25.0%)	9 (17.3%)	0	0
	Female	40	39 (97.5%)	9 (22.5%)	7 (17.5%)	9 (22.5%)	0	0
**Study region**	Gabes	25	24 (96.0%)	14 (56.0%)	13 (52.0%)	3 (12.0%)	0	0
	Medenine	40	39 (97.5%)	5 (12.5%)	5 (12.5%)	9 (22.5%)	0	0
	Tataouine	27	25 (92.6%)	4 (14.8%)	2 (7.4%)	6 (22.%)	0	0
**Total**		92	88 (95.6%)	23 (25.0%)	20 (21.7%)	18 (19.5%)	0	0

HAV seroprevalence in patients with acute hepatitis was 95,6%, ranging from 94,6% in patients under 10 years-old to 100% in patients over 30 years-old; there was no difference according to governorate. Twenty-three patients tested also positive for anti-HAV IgM with detectable HAV RNA in 20/23 of these. In 3 patients aged 11, 25 and 36 years, HAV RNA was undetectable, and anti-HAV IgG avidity index was high, both results indicating past HAV infection. HAV RNA was detectable in the remaining 20 patients. Acute HAV infections thus represented 21,7% of acute hepatitis etiology in this series. Patients with acute HAV infection were younger than patients with other hepatitis causes (9,8 years vs. 20,4 years, p = 0,004), and 65% were male. Most acute HAV infections were observed in Gabès where HAV infections represented 13/25 (52%) of hepatitis etiology. The proportion of acute HAV in the two other governorates was lower: 5/40 (12,5%) and 2/27 (7,4%) in Medenine and Tataouine, respectively (p<0.0001) Table 2, S2 Table.

The number of admissions for hepatitis peaked in the period from September to December with 39 cases among 92 (42.4%), and HAV was the cause identified in 17/39 cases (43.6%); 34/92 admissions (36.9%) were recorded between January and April with only 3 HAV cases (8.8%); and 19 admissions were recorded between May and August with no HAV cases.

Considering the 18 patients with anti-HEV IgG-positive results, all had markers of past HAV infection, including the 2 adults aged 25 and 36 years, who presented with detectable anti-HAV IgM related to immune activation as evidenced by undetectable HAV viremia and elevated anti-HAV IgG avidity index.

### HAV sequences analysis

**Fig 1:**
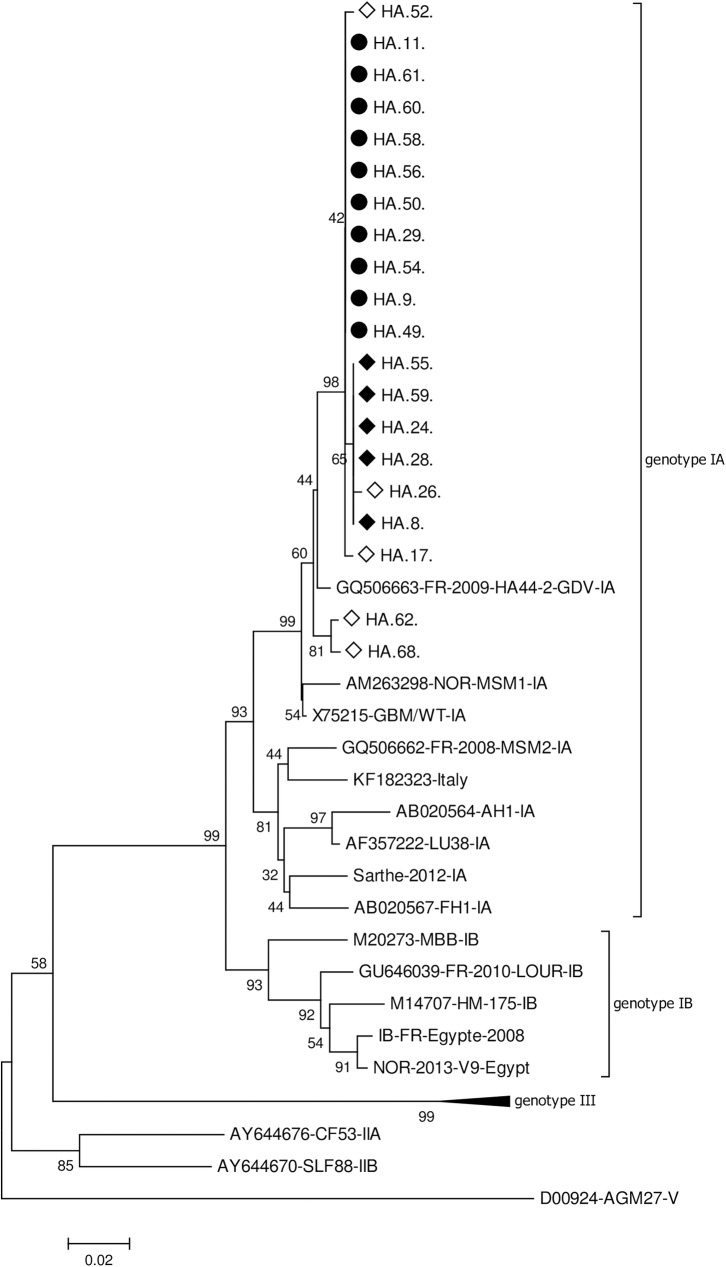
Phylogenetic analysis of VP1/2A sequences (384 nt-long) recovered from Tunisia. Reference sequences are indicated with their GenBank accession number. Sequences from this study are indicated by the name of the isolate (GenBank accession numbers KY172049 to KY172068). ● cluster from Gabès, ◆ cluster from Medenine, ◇ unique strains

Nucleotide sequences were obtained for the 20 HAV RNA-positive cases of the acute hepatitis group (GenBank accession numbers KY172049 to KY172068). Estimates of genetic diversity were conducted on MEGA6 and included 395 positions. The genetic diversity of the 20 HAV sequences was low with a mean Kimura 2-parameter distance of 0.005. Phylogenetic analysis identified 2 clusters and 5 unique sequences, all belonging to genotype IA (Fig 1).

A first cluster of 10 sequences included most sequences from Gabès (10/13), isolated both in autumn 2014 and autumn 2015. A second cluster of five sequences included all the sequences from Medenine. Unique strains were responsible for the remaining 3 cases from Gabès, diagnosed between January and May 2015, and for the 2 cases from Tataouine.

Additional, phylogenetic analyses conducted with previously published sequences from North and Central Tunisia [12] identified a specific clustering of Southern Tunisian sequences. However, within a common analyzable fragment of 274nt a strict homology was observed between published sequences and 2 unique strains from Gabès isolated from adults: HA62 (GenBank KY172065) with KP091382 isolated in 2013 in Monastir, and HA68 (GenBank KY172066) with KP091362 isolated in Tunis in 2012. The remaining 18 sequences also showed a high homology with sequences recovered in Tunis and Monastir in 2012–2013 (KP091378 and KP091359).

## Discussion

The present study addresses for the first time HAV and HEV seroprevalence in the Southern part of Tunisia among two specific groups of patients, pregnant women and acute hepatitis patients, and confirms the low HEV endemicity of the country while high HAV endemicity is observed in this southern region with very high seroprevalence by age 10 and frequent acute HAV cases among symptomatic patients.

Regarding HEV, we observed a rather low seroprevalence among pregnant women from Medenine (5.1%) with no relation to age, while higher seroprevalence (19,5%), related to age, was observed among acute hepatitis patients from all 3 governorates. This difference could be explained in part by a different age structure of these two populations. The Wantai assay used in our study has been reported to present enhanced specificity and sensitivity compared to other immunoassays [13] and comparison with previously reported data in Tunisia is thus turned difficult. A systematic review on HEV prevalence in Africa reports seroprevalence rates ranging from 0% among young village residents in Gabon to 84.3% among pregnant women in Egypt [14]. Focusing on the North African area, data from Egypt, Morocco and Tunisia are available. Generally, reported seroprevalences are much higher in Egypt than in Morocco or Tunisia, where reported seroprevalence rates do not exceed 46% in older adults. In Northern Tunisian regions, by using the Globe Diagnostics assay, reported seroprevalences were 12;1% among pregnant women [7], 28.9% among polytransfused patients [7], 10.2% among hemodialysis patients [6] and 4,5% among blood donors [6]. A similar low prevalence of 5.4% was reported among blood donors by using the Genelabs Diagnostics assay [8]. By using a more sensitive assay, our study thus suggests that Southern Tunisia is indeed a low endemicity region for HEV. This is further suggested when seroprevalence among acute hepatitis patients (19;5%) is compared to that reported from Egypt (30,9%) by using the Adaltis assay which is less sensitive than the Wantai assay [15]. The absence of acute HEV cases among symptomatic patients further confirms the low HEV endemicity. Besides, the low IgM index and negative viremia in 3 pregnant women with normal ALT may correspond either to a late diagnosis of an asymptomatic infection or to false positive IgM results. HEV is transmitted from humans to humans by feces-contaminated water but HEV is also transmitted from animals to humans by meat consumption, by contact with infected animals and probably also by the environment contaminated by animal feces [16]. The Tunisian food culture does not include pork and there is no pig farming. However, boars and camels, also potential HEV reservoirs are common, and seafood, potentially contaminated by environmental HEV, is consumed in coastal areas. The low HEV prevalence evidence by our study suggests that both human and zoonotic HEV are not endemic in Southern Tunisia.

By contrast, HAV seroprevalence exceeded 90% by age 5 (21/23) or 10 (35/37) and acute HAV infections represented 21.7% of hepatitis etiologies. Tunisia, and neighboring North African countries are reported to have an high to intermediate levels of anti-HAV seroprevalence (≥50% of immunity by age 15) [2, 3]. Seroprevalence is shown to increase with older age and lower social class and to vary between urban and rural settings. For example; in Central Tunisia, 21.3% of school children living in urban areas and 87.7% of those living in rural had antibodies to HAV by age 10 in 2002 [17]. About 15 years later, similar figures are still observed in rural areas from Southern Tunisia, as shown here. Due to particularly high HAV seroprevalence among acute hepatitis patients from the 3 governorates, ranging from 92.6% in Tataouine to 97.5% in Medenine, no significant difference was observed according to geographical region. However, acute HAV infection was rare in Tataouine (7.4%) and very frequent in Gabès (52%), reflecting different HAV exposure between a semi-desert area and a coastal region. In line with this finding, the proportion of HAV cases among hepatitis patients observed in Gabès is similar to the 51.5% reported from the coastal regions of Monastir and Sousse [18]. Of note, the negative viremia and high avidity IgG index in 3/23 IgM-positive patients (13%) corresponding to late IgM or to immune activation, confirms how tricky could be IgM interpretation [10].

HAV genotype IA was recovered from all HAV cases. This finding was expected, since IA is the most frequently reported subtype in Northern Tunisia, followed by IB [12] [19]. HAV isolates described here cluster with previously reported isolated from Tunisia, reviewed in [12]. This clustering allows the definition of a geographical Tunisian signature, as published [20] and indicates the continuous circulation of these strains in Tunisia. Of note, the same strain was identified in patients from Gabès one year apart. This persistence may be related to silent person-to-person transmission, but more likely to environmental contamination, as published [21].

The dramatic difference between HAV and HEV seroprevalences reported here certainly reflect different spreading modes related to differences in viral infectivity, stability and transmissibility. Furthermore, our results suggest not only that HEV infection is less common than HAV, but also that infection is usually asymptomatic. However, our study has several limitations, though it is the first to provide data from Southern Tunisia. First, it’s primarily a descriptive study in selected populations, with rather low numbers, that may not represent the real picture of the entire population of this region. Secondly, no survey was implemented to investigate HAV or HEV risk factors, and notably for HAV the transmission mode was not investigated. For this specific point, we can only extrapolate environmental data from other coastal regions and assume that there is also an environmental contamination and an environmental reservoir for HAV in this region.

In terms of public health policy, the very high HAV prevalence found in this study stresses the need for improved environmental and personal sanitation and hygiene that can induce marked reduction in fecal-oral virus transmission, without a specific vaccine strategy [2]. Indeed, water, sanitation and hygiene (WASH) are critical in the prevention and care for all of the 17 neglected tropical diseases, which include HAV, scheduled by the World Health Organization (WHO) for intensified control or elimination by 2020 (http://www.who.int/water_sanitation_health/publications/wash-and-ntd-strategy/en/).

Universal immunization programs in early childhood (1–2 years-old)) may also reduce dramatically the incidence of hepatitis A, particularly in areas that are transitioning to lower levels of transmission resulting in an increasing proportion of susceptible adolescents and adults, often in urban areas or higher socioeconomic classes. Countries that have implemented universal immunization programs (Israel, Italy, United States, Argentina, …) have demonstrated a successful impact on HAV incidence. However, WHO guidance on HAV vaccines emphasizes the need to assess the cost-benefit and sustainability of these prevention strategies [2].

## Conclusion

Our study has addressed for the first time HAV and HEV burden in specific populations from Southern Tunisia. A still high endemicity for HAV infection is highlighted in this region and additional Tunisian HAV sequences were provided.

## Supporting information

S1 Table Crude data for pregnant women. (XLSX)

S2 Table Crude data for patients with acute hepatitis. (XLSX)
